# Estimation of ground-level PM_2.5_ concentration using MODIS AOD and corrected regression model over Beijing, China

**DOI:** 10.1371/journal.pone.0240430

**Published:** 2020-10-13

**Authors:** Xinghan Xu, Chengkun Zhang

**Affiliations:** 1 Department of Environmental Engineering, Kyoto University, Kyoto, Japan; 2 Faculty of Electronic Information and Electrical Engineering, Dalian University of Technology, Dalian, China; University of Birmingham, UNITED KINGDOM

## Abstract

To establish a new model for estimating ground-level PM_2.5_ concentration over Beijing, China, the relationship between aerosol optical depth (AOD) and ground-level PM_2.5_ concentration was derived and analysed firstly. Boundary layer height (BLH) and relative humidity (RH) were shown to be two major factors influencing the relationship between AOD and ground-level PM_2.5_ concentration. Thus, they are used to correct MODIS AOD to enhance the correlation between MODIS AOD and PM_2.5_. When using corrected MODIS AOD for modelling, the correlation between MODIS AOD and PM_2.5_ was improved significantly. Then, normalized difference vegetation index (NDVI), surface temperature (ST) and surface wind speed (SPD) were introduced as auxiliary variables to further improve the performance of the corrected regression model. The seasonal and annual average distribution of PM_2.5_ concentration over Beijing from 2014 to 2016 were mapped for intuitively analysing. Those can be regarded as important references for monitoring the ground-level PM_2.5_ concentration distribution. It is obviously that the PM_2.5_ concentration distribution over Beijing revealed the trend of “southeast high and northwest low”, and showed a significant decrease in annual average PM_2.5_ concentration from 2014 to 2016.

## Introduction

With vibrant development of economy and rapid improvement of industry, environmental issues have gradually become a focus of attention. In recent years, the problem of haze has become outstanding, and the main reason for this is the absorption and scattering of PM_2.5_ (particulate matter with aerodynamic diameters less than or equal to 2.5 *μ*m) in visible light intensity. In addition to affecting the human daily life and economical production, PM_2.5_ also contributes to chronic respiratory, pulmonary, cardiovascular, and other human diseases [1, 2]. The reduction of PM_2.5_ concentration has been a matter of great urgency all over the world.

The real-time monitoring of PM_2.5_ concentration plays a vital role in PM_2.5_ concentration reduction and environmental protection. Monitoring methods of PM_2.5_ concentration can be generally divided into two categories: ground stations monitoring and satellite remote sensing image estimating [3]. The former obtains environmental pollution information by setting up monitoring stations on the earth surface. This category can provide accurate monitoring of PM_2.5_ concentration, but the spatial distribution of monitoring stations is uneven and the equipment is expensive, making it difficult to obtain a wide range distribution of PM_2.5_ concentration. The latter can acquire continuous, large scale atmospheric aerosol information through remote sensing data, which can be converted to the PM_2.5_ concentration and distribution of the earth surface [4–6]. This category is more comprehensive and can greatly reduce the cost of research. So building a high-precision regression model between satellite based atmospheric aerosol information and ground measured PM_2.5_ concentration has great practical significance.

Griggs et al. discovered that ERTS-1 satellite data provided the possibility of monitoring atmospheric AOD [7]. Meanwhile, the monotonic linear relationship between the radiation values of infrared and visible channels and AOD was confirmed theoretically. Since Moderate Resolution Imaging Spectroradiometer (MODIS) sensors were put into use, AOD inversion algorithms and products have been greatly developed. It pushed the aerosol inversion research from ocean to land which supports most human activities. Dark target (DB) and Deep blue (DT) algorithms have gradually become mature AOD inversion algorithms over land through continuous improvement and development. Butt et al. adopted the DB algorithm AOD products of the Terra and Aqua satellites from 2000 to 2013 to analyse changes of AOD over Arabia, and concluded that the Aqua satellite’s AOD data were closer to the AOD results measured by ground stations than the Terra satellite’s AOD data [8]. Bilal et al. utilized AOD data from Aerosol Robotic Network (AERONET) level 2.0 cloud screening and quality control data to evaluate the static Normalized Difference Vegetation Index (NDVI) pixel selection criteria of AOD which based on the combination of the DB, DT, and DT and DB (DTB) algorithms. The experimental results revealed that the proposed dynamic NDVI pixel selection criteria could improve the performance of DTB products and reduce uncertainty [9].

With the shrinkage of particulate matter index from PM_10_ to PM_2.5_ and new development of AOD products, researchers have exploited AOD data to estimate PM_2.5_ concentration. The main approaches generally include statistical models and physical-chemical models. Compared to the physical-chemical models which require complex analysis of light transmission process in atmosphere and are difficult to generalize, statistical ones can use a regression model fitted by the relationship between AOD and PM_2.5_ concentration to approximately estimate the PM_2.5_ concentration.

Statistical models can be divided into the linear regression model, mixed effect model, geographically weighted regression model (GWR), and some others. On the basis of measured AOD and mixed layer height data, Schäfer et al. derived the extinction coefficient of aerosol, and acquired the extinction efficiency of aerosol through a linear regression model [10]. The experimental results showed that the extinction coefficient of aerosol was significantly related to the surface PM concentration in winter conditions. To study the correlations between PM_2.5_ concentration and AOD in North China, Xin et al. established linear regression functions between ground-level PM_2.5_ concentration and ground-observed AOD and MODIS AOD, respectively [11]. The results indicated that the linear regression functions of different seasons are significantly different and this is because the aerosol types were affected by seasonal variations. Tai et al. analysed the relationship between PM_2.5_ and different meteorological variables using the multivariate regression model (MLR) from 1998 to 2008 in adjacent areas of the United States [12]. The experiments showed that the distribution of PM_2.5_ are influenced by disturbances of wind patterns due to climate change.

Lee et al. proposed a mixed effect model, which fully exploited remote sensing data to establish the relationships between daily PM_2.5_ concentration and AOD in the New Zealand region and suggested the daily relations between AOD and PM_2.5_ concentration through the statistical method [13]. Xie et al. developed a mixed effect model to invert daily PM_2.5_ concentration in Beijing, which taken the diurnal variation relationship of AOD and PM_2.5_ concentration into account [14]. This research concluded that high-resolution daily PM_2.5_ concentration maps can contribute to assessment of short-term and long-term PM_2.5_ exposure. Zheng et al. put forward a linear mixed effect model that integrated AOD measurement, meteorological parameters, and NO_2_ column density for the estimation of PM_2.5_ concentration in Beijing, Tianjin and Hebei, the Yangtze River Delta, and the Pearl River Delta [15]. As shown in the experimental results, the combination of different factors contributed to the accuracy of model, which provided the possibility of assessing PM_2.5_ concentration in areas with different pollution levels.

You et al. adopted the GWR model to estimate the concentration of PM_2.5_ concentration in China and introduced meteorological features as auxiliary variables into the model to improve the accuracy [16]. In the experiments, cross validated R^2^ reached 0.79. The experimental results demonstrated that the proposed approach could evaluate a large area of PM_2.5_ concentration distribution. Guo et al. proposed a geographic and temporal weighted regression model (GTWR), which took both the temporal and spatial variability into consideration [17]. The AOD measurements, meteorological factors, and land use variables were also employed for fitting different seasons. The experiments indicated that GTWR was superior to MLR and GWR but that it was influenced by the sampling frequency of PM_2.5_ data [17]. Continuous daily PM_2.5_ observations could improve the performance of the model.

The aforementioned methods, such as MLR, mixed effects mode, GWR, GTWR, established the empirical linear relationship between PM_2.5_ concentration and satellite based AOD, and successfully mapped the PM_2.5_ concentration distribution of the study area. However, adequate analysis of how the relevant factors, such as boundary layer height (BLH) and relative humidity (RH), effect the PM_2.5_ concentration from a theoretical point of view is also essential for estimation of PM_2.5_ concentration distribution.

In this work, we first analyse the relationship between ground-level PM_2.5_ concentration and AOD, BLH, RH, and get the conclusion that BLH and RH can effectively correct the relationship between AOD and PM_2.5_ concentration. Thereby, three kinds of more theoretical AOD-PM_2.5_ regression models are established for PM_2.5_ concentration estimation, which are more interpretable. Then, NDVI, ST and SPD are introduced as auxiliary variables to further improve the performance of the corrected regression model. The modified AOD-PM_2.5_ concentration regression models were established separately according to the season and used to estimate the seasonal average PM_2.5_ concentration over Beijing, China during 2014 to 2016. The seasonal and annual PM_2.5_ concentration distribution over Beijing were mapped using the established models for better analysing the geographic distribution and annual changes of PM_2.5_ concentration.

## Materials and methods

Beijing, located at 39.4°-41.6° N, 115.7°-117.4° E, is an important economic and political center of China. With the rapid development of the economy, Beijing has become one of the cities heavily affected by haze, especially in spring and winter. PM_2.5_ has a serious impact on urban traffic and people’s life. In this section, MODIS AOD and NDVI products, station-measured PM_2.5_ data, four kinds of meteorological data are described in this section (seen in Table 1).

**Table 1:** Descriptive statistics of PM_2.5_ measurements, MODIS AOD product, NDVI, and meteorological data during the study period (2014 to 2016).

Variable	Unit	Temporal resolution	Spatial resolution	Source
AOD	Unitless	1 day	10km	MODIS
NDVI	Unitless	monthly	1km	MODIS
PM_2.5_	*μ*g/m^3^	1 hour	15 stations	CEMC
RH	%	6 hour	0.125°	ECMWF
BLH	m	3 hour	0.125°	ECMWF
ST	°C	3 hour	0.125°	ECMWF
SPD	m/s	3 hour	0.125°	ECMWF

### PM_2.5_ measurements

**Fig 1:**
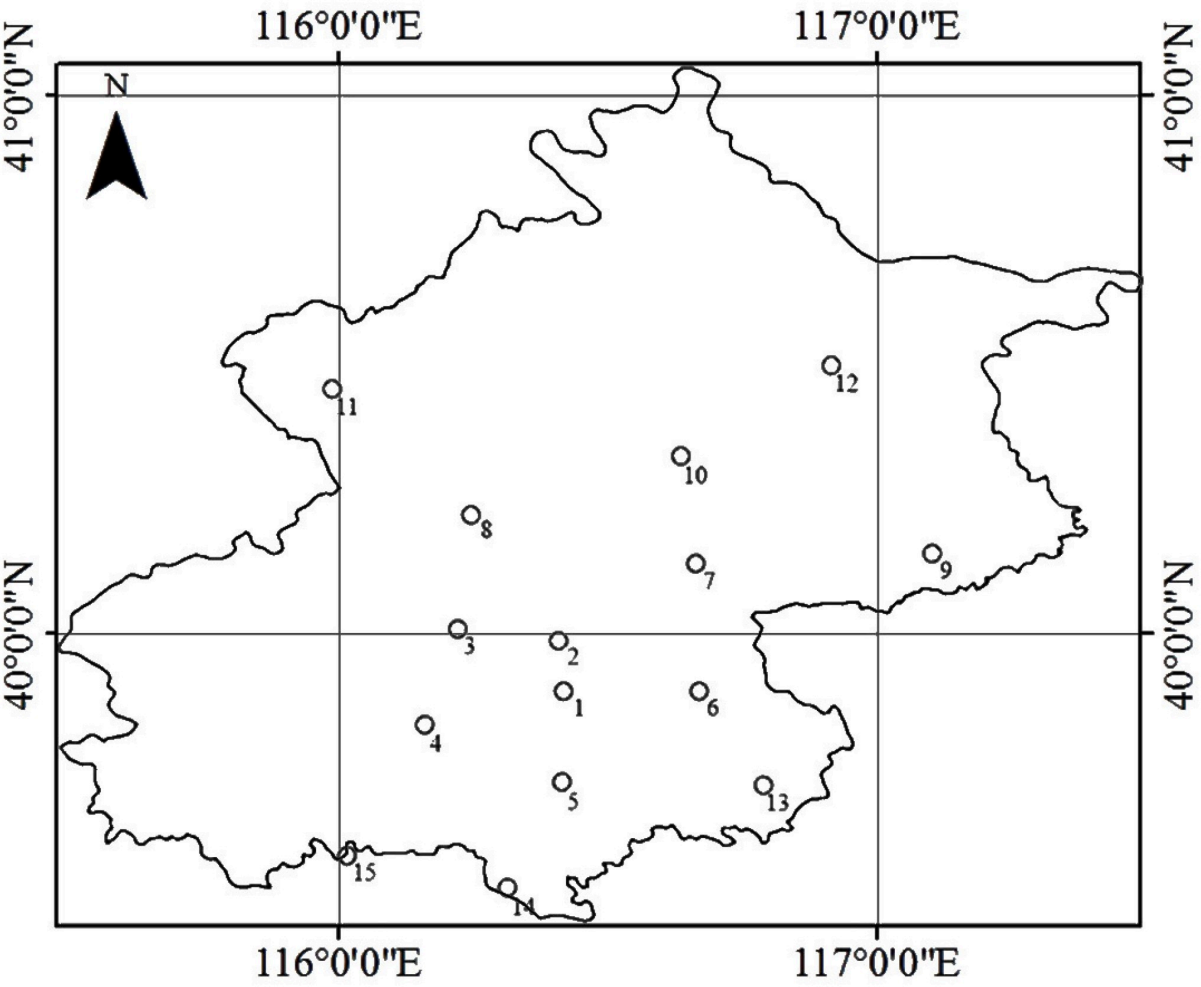
The location of 15 air quality monitoring stations in Beijing.

Hourly station-measured PM_2.5_ concentration data observed at 15 uniformly-distributed air quality monitoring station in Beijing, which were collected from the Beijing Municipal Environmental Monitoring Center (http://zx.bjmemc.com.cn/) [18, 19], were used for the experiments. The location of all the 15 air quality monitoring stations in Beijing is shown in Fig 1. and the geographical coordinates are listed in S1 Table. We averaged the measured PM_2.5_ concentration between 13:00 pm and 14:00 pm local time to match the overpass time of the MODIS Aqua satellite (about 13:30 pm local time). The study period was from January 1, 2014, to December 31, 2016, spanning a total of 1096 days.

### MODIS AOD and NDVI products

MODIS onboard the NASA Aqua satellite has been in operation since 2002. It provides retrieval products of aerosol and cloud properties with nearly daily global coverage. The MODIS data used in this study were the MYD04 Level 2 aerosol products with a resolution of 10 km and MYD13 monthly NDVI products with a resolution of 1 km [9]. The overpass time of the MODIS Aqua satellite is about 13:30 pm local time. The MODIS DT aerosol algorithms produce two separate products with different resolutions, with spatial resolutions of 3km and 10km respectively. However, it performs unsatisfactorily in urban regions. The DB algorithm performs better than the DT algorithm over urban areas, and it can provide better land coverage over both dark and bright surfaces, which is more reasonable for PM_2.5_ concentration distribution research in Beijing [20–22].

### ECMEF ERA interim meteorological data

The ERA-Interim data provided by the European Centre for Medium-Range Weather Forecasts (ECMWF) are real-time updated global atmospheric reanalysis data (https://www.ecmwf.int/en/forecasts/datasets/reanalysis-datasets/era-interim). The meteorological parameters used in this study are BLH and RH at 100m vertical direction, surface temperature and wind speed at 14:00 pm local time [23]. The spatial resolution of the ERA-Interim reanalysis data used in this study was approximately 0.125°.

### AOD-PM_2.5_ corrected regression model

Because some parameters used to describe atmospheric physical conditions, such as air pressure, atmospheric temperature, and atmospheric humidity, change much more in the vertical direction than the horizontal direction [24], it is often assumed that the atmosphere has a structure in which the horizontal direction is uniform and the vertical direction is layered. When considering a single homogeneous atmospheric layer containing spherical aerosol particles, the mass concentration at the surface can be represented as

$$\begin{array}{c} \text{PM}=\frac{4}{3}\pi \rho \int r^{3}n_{dry}\left(r\right)dr, \end{array}$$ (1)

in which *ρ* denotes the density of aerosol particles (g/m^3^) and *n*_*dry*_(*r*) denotes the particle size distribution spectrum under dry conditions. Generally, AOD is the integral of the extinction coefficient of aerosol particles in the vertical direction of the atmosphere with aerosol scale height *H* [25]

$$\begin{array}{c} \text{AOD}\left(\lambda \right)=\int_{0}^{H}\sigma_{ext,amb}\left(\lambda \right)dz, \end{array}$$ (2)

where *σ*_*ext*,*amb*_ denotes the aerosol extinction coefficient under ambient conditions. It can be expressed as $\sigma_{ext,amb}\left(\lambda \right)=\int_{0}^{\infty }\pi Q_{ext,amb}\left(r,\lambda \right)n_{amb}\left(r\right)r^{2}dr$, where *Q*_*ext*,*amb*_ denotes the particulate matter extinction efficiency under ambient conditions and *n*_*amb*_(*r*) denotes the particle size distribution under ambient conditions. Thus, at a given wavelength λ, the aerosol optical thickness from the ground to the height *H* can be expressed as [26]

$$\begin{array}{c} \text{AOD}=\pi \int_{0}^{H}\int_{0}^{\infty }Q_{ext,amb}\left(r\right)n_{amb}\left(r\right)r^{2}drdz. \end{array}$$ (3)

As we all know, different aerosol particles have different hygroscopic properties, e.g., water-soluble particles and organic aerosol particles have distinct hygroscopic properties. Thus, aerosol particles with the same mass concentration but different composition show different extinction characteristics under different humidity conditions. That is to say, the correlation between the extinction coefficient and the mass concentration changes with humidity. Therefore, to reduce the uncertainty introduced by the hygroscopic growth of the particles, it is necessary to convert the AOD obtained by satellite remote sensing into the mass concentration of dry particles.

A hygroscopic growth factor *f*(RH), which represents the ratio between these (size-distribution integrated) extinction efficiencies, is required to convert particulate matter extinction efficiency under ambient conditions to under dry conditions. Then, Eq (3) can be converted to the following [26, 27]

$$\begin{array}{c} \text{AOD}=\pi f(\text{RH})\int_{0}^{H}\int_{0}^{\infty }Q_{ext,dry}\left(r\right)n_{amb}\left(r\right)r^{2}drdz. \end{array}$$ (4)

Furthermore, to rewrite the above formula, the size-distribution integrated extinction efficiency 〈*Q*_*ext*_〉 and effective radius *r*_*eff*_ can be introduced:

$$\begin{array}{c} \langle Q_{ext}\rangle =\frac{\int Q_{ext}\left(r\right)n\left(r\right)r^{2}dr}{\int n\left(r\right)r^{2}dr}, \end{array}$$ (5)

$$\begin{array}{c} r_{eff}=\frac{\int n\left(r\right)r^{3}\left(r\right)dr}{\int n\left(r\right)r^{2}\left(r\right)dr}. \end{array}$$ (6)

Hence, the relationship between AOD and near-surface PM mass concentration is finally derived as follows [25, 28]

$$\begin{array}{c} \text{AOD}=\text{PM}\cdot H\cdot f(\text{RH})\frac{3\langle Q_{ext,dry}\rangle }{4\rho r_{eff}}. \end{array}$$ (7)

As the aerosol scale height *H* is an assumed equivalent height under ideal conditions, it is difficult to measure directly when the near-surface aerosol extinction coefficient is unknown. In the planetary boundary layer, the obvious turbulent motion causes strong atmospheric mixing, resulting in a uniform vertical distribution of aerosols. In practice, the BLH has a similar physical meaning to the aerosol scale height *H* and is often used instead of *H*.

According to the above relationship between AOD and PM, it can be inferred that if the AOD is corrected by the factors BLH and *f*(RH), the corrected AOD should be expected to obtain a better correlation with PM.

Here, a time-space matched MODIS AOD-PM_2.5_ data pair and a corresponding BLH and *f*(RH) were used to construct different linear regression models in different seasons [26]. There were 4 types of models:

$$\begin{array}{c} I:\text{AOD}=a_{1}+b_{1}PM_{2.5}, \end{array}$$ (8)

$$\begin{array}{c} \text{II}:\text{AOD}/\text{BLH}=a_{2}+b_{2}PM_{2.5}, \end{array}$$ (9)

$$\begin{array}{c} \text{III}:\text{AOD}/(f(\text{RH}))=a_{3}+b_{3}PM_{2.5}, \end{array}$$ (10)

$$\begin{array}{c} \text{IV}:\text{AOD}/(\text{BLH}\;\cdot f(\text{RH}))=a_{4}+b_{4}PM_{2.5}. \end{array}$$ (11)

where a and b are model regression coefficients. Eq (8) represents a simple, original regression model, Eq (9) represents a model corrected by BLH, Eq (10) represents a model corrected by RH, and Eq (11) represents a model jointly corrected by BLH and RH.

In this study, NDVI, ST and SPD are introduced as auxiliary variables to further improve the performance of the corrected regression model for estimating ground-level PM_2.5_ concentration. 4 types of modified corrected regression models can be described as

$$\begin{array}{c} V:\text{AOD}=a_{1}+b_{1}PM_{2.5}+c_{1}\text{NDVI}+d_{1}\text{ST}+e_{1}\text{SPD}, \end{array}$$ (12)

$$\begin{array}{c} \text{VI}:\text{AOD}/\text{BLH}=a_{2}+b_{2}PM_{2.5}+c_{2}\text{NDVI}+d_{2}\text{ST}+e_{2}\text{SPD}, \end{array}$$ (13)

$$\begin{array}{c} \text{VII}:\text{AOD}/(f(\text{RH}))=a_{3}+b_{3}PM_{2.5}+c_{3}\text{NDVI}+d_{3}\text{ST}+e_{3}\text{SPD}, \end{array}$$ (14)

$$\begin{array}{c} \text{VIII}:\text{AOD}/(\text{BLH}\;\cdot f(\text{RH}))=a_{4}+b_{4}PM_{2.5}+c_{4}\text{NDVI}+d_{4}\text{ST}+e_{4}\text{SPD}. \end{array}$$ (15)

Those modified corrected regression models are fitted separately according to the season and used to estimate the seasonal average PM_2.5_ concentration over Beijing, China during 2014 to 2016. For model evaluation, we assessed the model performance by calculating the coefficient of determination R^2^ and root mean squared error (RMSE) between estimated and measured PM_2.5_ concentration in different seasons:

$$\begin{array}{c} R^{2}=1-\frac{\sum_{i=1}^{n}{\left(y_{i}-f_{i}\right)}^{2}}{\sum_{i=1}^{n}{\left(y_{i}-{\bar{y}}_{i}\right)}^{2}}, \end{array}$$ (16)

$$\begin{array}{c} \text{RMSE}=\sqrt{\frac{1}{N}\sum_{i=1}^{n}{\left(y_{i}-f_{i}\right)}^{2}}, \end{array}$$ (17)

where *f*_*i*_ and *y*_*i*_ are the predicted PM_2.5_ and the measured PM_2.5_ concentration of the *i*th sample, respectively, and *N* is the total number of AOD-PM_2.5_ pair samples.

**Fig 2:**
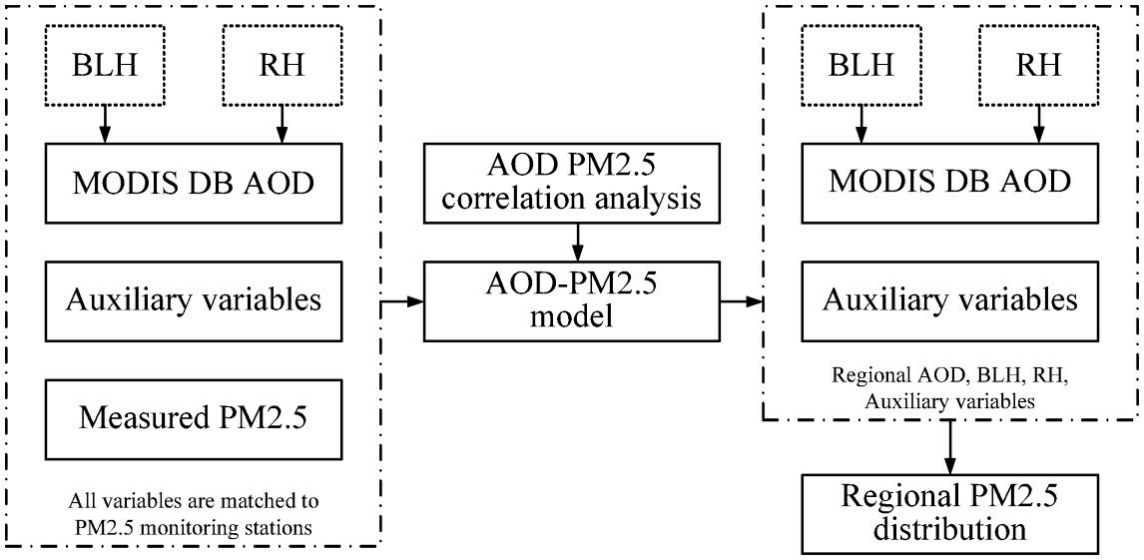
Flow diagram for developing the modified corrected AOD-PM_2.5_ model in Beijing.

Fig 2 shows the flow diagram of the development process of the modified corrected AOD-PM_2.5_ model over Beijing. Firstly, BLH, RH, ST and SPD are interpolated to the same resolution as MODIS DB AOD by means of bilinear interpolation. NDVI is resized to the same resolution as MODIS DB AOD by means of nearest neighbor. Then, all variables located at 15 air quality monitoring stations are used to construct a AOD-PM_2.5_ modified regression model according to the season in every year. Finally, the seasonal and annual PM_2.5_ concentration distribution map is calculated by the AOD-PM_2.5_ modified regression model, which can directly reflect the PM_2.5_ distribution of Beijing.

## Results and discussions

In our study, the correlation between estimated and measured PM_2.5_ concentration using aforementioned models is evaluated. R^2^ between estimated and measured PM_2.5_ concentration obtained by different models according to the season during 2014-2016 is tabulated in Table 2. *f*(RH) is defined empirically as *f*(RH) = 1/(1 − RH/100).

**Table 2:** R^2^ between estimated and measured PM_2.5_ concentration obtained by different models according to the season during 2014-2016.

Model	I	II	III	IV	V	VI	VII	VIII
Season								
Spring, 2014	0.72	0.75	0.73	0.75	0.75	0.76	0.75	**0.77**
Summer, 2014	0.66	0.64	0.68	0.65	0.68	0.68	0.68	**0.69**
Autumn, 2014	0.72	0.81	0.72	0.81	0.73	**0.82**	0.74	0.81
Winter, 2014	0.68	0.69	0.68	0.69	**0.70**	0.69	0.69	0.69
Spring, 2015	0.69	0.71	0.69	0.71	0.70	0.73	0.70	**0.74**
Summer, 2015	0.68	0.65	0.67	0.64	0.72	**0.75**	0.71	0.74
Autumn, 2015	0.73	0.81	0.73	0.81	0.74	**0.82**	0.73	0.81
Winter, 2015	0.69	0.69	0.68	0.69	**0.73**	0.73^1^	0.72	0.73^1^
Spring, 2016	0.76	0.82	0.76	0.82	0.76	**0.84**	0.77	0.83
Summer, 2016	0.62	0.58	0.73	0.60	0.63	0.61	**0.74**	0.63
Autumn, 2016	0.70	0.79	0.70	0.79	0.72	**0.80**	0.72	0.79
Winter, 2016	0.71	0.80	0.70	0.80	0.70	0.81	0.70	**0.82**

^1^ In Autumn of 2015, the R^2^ of model V is higher than that of model VI and VIII when using more scientific numbers.

In Table 2, the R^2^ of model I can be interpreted as the correlation between measured PM_2.5_ concentration and MODIS DB AOD. Generally, in spring and autumn, the estimated PM_2.5_ concentration had closer correlation with the measured PM_2.5_ concentration. This may be a result of more uniform near-surface aerosol composition, as well as lower BLH, which limits aerosol diffusion in Beijing during spring and autumn.

According to Eq (7), BLH and *f*(RH) corrections could improve the AOD-PM_2.5_ correlation theoretically. The BLH corrected model was established on the basis of this. In Table 2, The R^2^ of model II can be interpreted as the correlation between measured PM_2.5_ concentration and BLH corrected AOD. Model correlations were affected differently in different seasons. The R^2^ in spring had a large increase after BLH correction, from 0.72 to 0.75 in 2014, from 0.69 to 0.71 in 2015, from 0.76 to 0.82. The more obvious advantages of BLH correction appears in autumn. The R^2^ has about 0.08-0.09 increase in different years, and reaches 0.81 in 2014 and 2015, 0.79 in 2016, demonstrating the effectiveness of BLH correction in autumn. For winter, the BLH corrected model achieved a slight improvement in 2014 and 2015 and a significant improvement in 2016. However, the R^2^ of the BLH corrected model had a decrease in summer, which because higher BLH cannot limit aerosol diffusion, as well as the poor quality of MODIS AOD products in summer because of cirrocumulus and rainfall.

The R^2^ of model III can be interpreted as the correlation between measured PM_2.5_ concentration and RH corrected AOD. With RH correction separately (Model III), a obvious increase of R^2^ are appeared in summer, 2016, from 0.62 to 0.73. However, the results change little in other times, and the fluctuation range of R^2^ is within 0.02. When considering BLH and *f*(RH) correction simultaneously (as Model IV), the results are very close to Model II.

**Fig 3:**
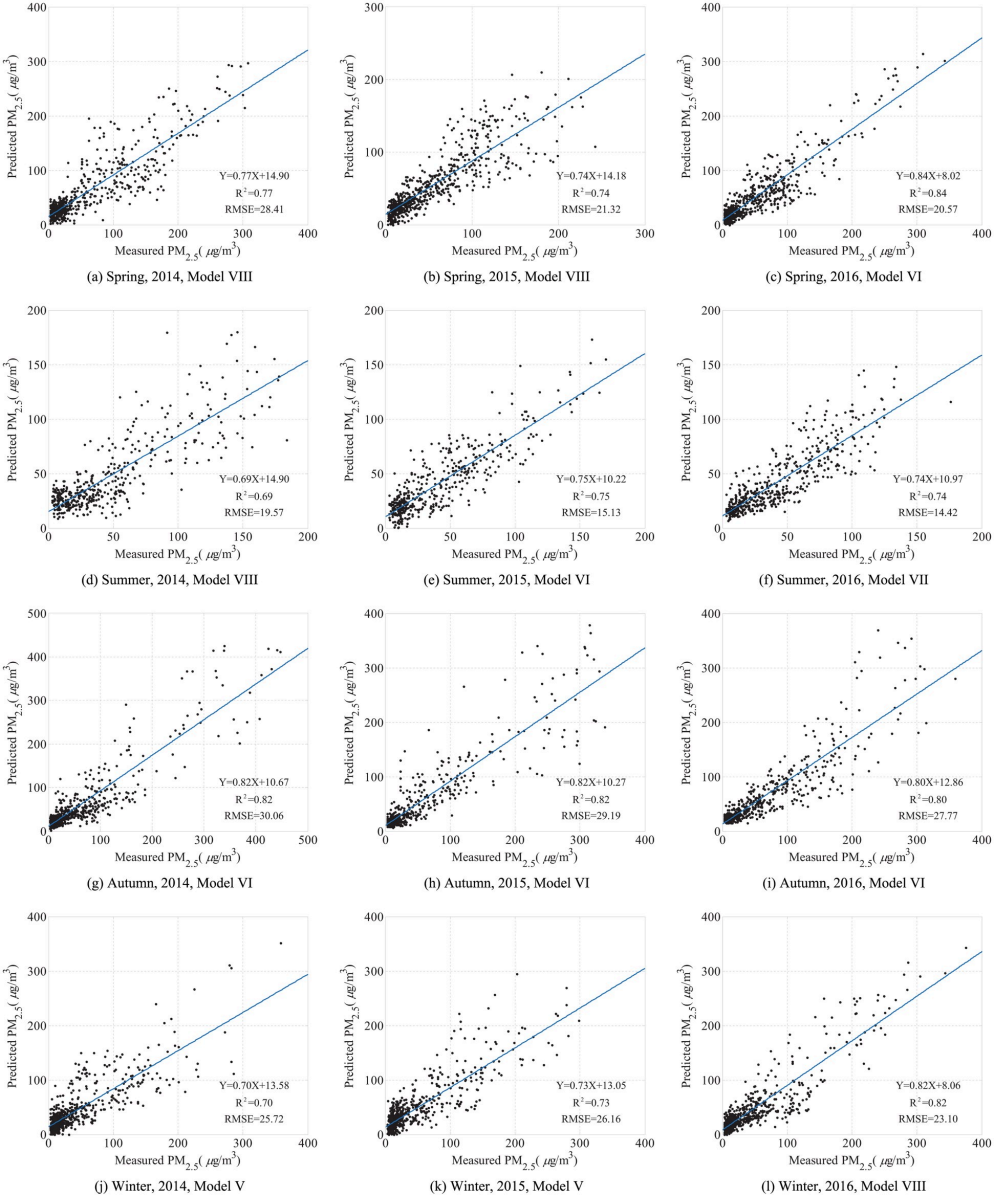
Scatter plots between estimated and measured PM_2.5_ concentration in different seasons during 2014 to 2016. (a) Spring, 2014, Model VIII. (b) Spring, 2015, Model VIII. (c) Spring, 2016, Model VI. (d) Summer, 2014, Model VIII. (e) Summer, 2015, Model VI. (f) Summer, 2016, Model VII. (g) Autumn, 2014, Model VI. (h) Autumn, 2015, Model VI. (i) Autumn, 2016, Model VI. (j) Winter, 2014, Model V. (k) Winter, 2015, Model V. (l) Winter, 2016, Model VIII.

When introduced NDVI, ST and SPD as auxiliary variables, the R^2^ of Model V-VIII in all seasons have about 2% increase correspondingly in most situations. Fig 3 show the scatter plots and regression results between estimated and measured PM_2.5_ concentration in the different seasons with the best results among all kinds of modified corrected models. In total, the correlation between estimated and measured PM_2.5_ concentration is more higher in spring and autumn. The R^2^ are 0.77, 0.74 and 0.84 in spring, 0.69, 0.75 and 0.74 in summer, 0.82, 0.82 and 0.80 in autumn, 0.70, 0.73 and 0.82 in winter during 2014 to 2016 respectively.

In our study, even though the complicated PM_2.5_ concentration distribution in Beijing, the modified corrected AOD-PM_2.5_ models achieved higher model fitting R^2^ values than those in previous studies, e.g., an observation-based algorithm that considers the effect of the main aerosol characteristics applied to the Beijing-Tianjin-Hebei region with R^2^ of 0.70 [3], the GWR model applied to the whole China mainland with an overall R^2^ of 0.64 [5], an improved model applied to the Beijing-Tianjin-Hebei region with an R^2^ of 0.77 [15], satellite-driven PM_2.5_ models with VIIRS nighttime data applied to the Beijing-Tianjin-Hebei region with R^2^ of 0.75 [19], and NAQPMS data incorporated to MODIS data applied to the Beijing-Tianjin-Hebei region from January to December 2017 with seasonal R^2^ values of 0.75, 0.62, 0.80, and 0.78 in the spring, summer, autumn, and winter, respectively [22].

Table 3 is comparison between estimated and measured seasonal average PM_2.5_ concentration of 15 monitoring stations from 2014 to 2016. Minor difference reflects the good performance of the established models in each season. Table 4. is the comparison of regional average PM_2.5_ concentration from 2014 to 2016 over Beijing. In term of interannual seasonal change, PM_2.5_ concentration in summer are more lower the other seasons because of a great deal of rainfall. PM_2.5_ concentration in spring and winter is general higher than other seasons because of low rainfall and home-heating. In term of annual change of PM_2.5_ concentration during 2014 to 2016, it witnesses a downward trend in the same season. The annual average PM_2.5_ concentration decrease from 75.1 *μ*g/m^3^ to 62.0 *μ*g/m^3^, about a 17% decrease, which confirms the effectiveness of a series of stringent clean air actions implemented by the Chinese government from 2013 to 2017 [29]. In 2016 winter, the PM_2.5_ concentration decreased to 59.4 *μ*g/m^3^, which is inspiring.

**Table 3:** The comparison between estimated and measured seasonal average PM_2.5_ concentration of 15 stations in Beijing from 2014 to 2016.(*μ*g/m^3^).

Year	Estimated	Measured
Spring, 2014	87.0	76.7
Summer, 2014	69.8	63.8
Autumn, 2014	87.7	89.0
Winter, 2014	83.2	76.7
Summer, 2015	69.7	65.3
Summer, 2015	60.3	51.0
Autumn, 2015	77.1	73.4
Winter, 2015	88.9	85.8
Summer, 2016	72.9	69.3
Summer, 2016	58.8	58.1
Autumn, 2016	77.2	86.0
Winter, 2016	67.5	66.9

**Table 4:** The comparison of average PM_2.5_ concentration from 2014 to 2016 in Beijing.(*μ*g/m^3^).

Year	2014	2015	2016
spring	79.3	67.7	69.1
summer	66.3	56.9	53.6
autumn	77.0	61.6	65.7
winter	77.7	77.9	59.4
Annual	75.1	66.0	62.0

**Fig 4:**
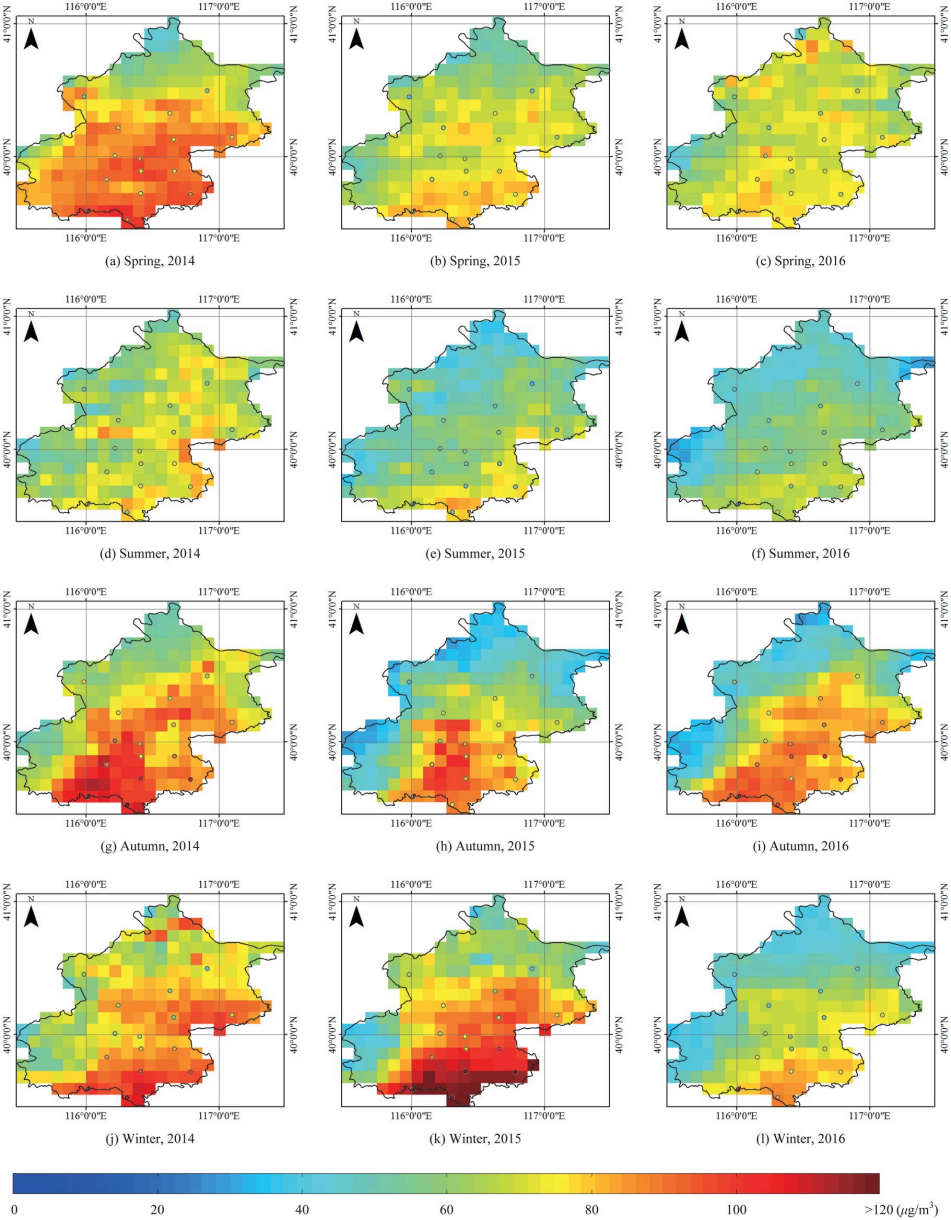
The seasonal average PM_2.5_ concentration distribution over Beijing during 2014 to 2016.

The spatial distribution of the seasonal and annual average PM_2.5_ concentration are mapped to investigate its spatial distribution characteristics over Beijing during 2014 to 2017, as shown in Fig 4. The measured average PM_2.5_ concentration is represented as a circle using the same color scheme. In Beijing, PM_2.5_ concentration spatial distribution reveals the trend of “southeast high and northwest low”. In spring, autumn and winter of 2014, autumn and winter of 2015, autumn of 2016, this trend is more obvious. PM_2.5_ concentration of the southeast of Beijing during these seasons are higher than 75 *μ*g/m^3^. This conforms to the urbanization level and population distribution in Beijing. Human activities directly affect the PM_2.5_ concentration.

**Fig 5:**
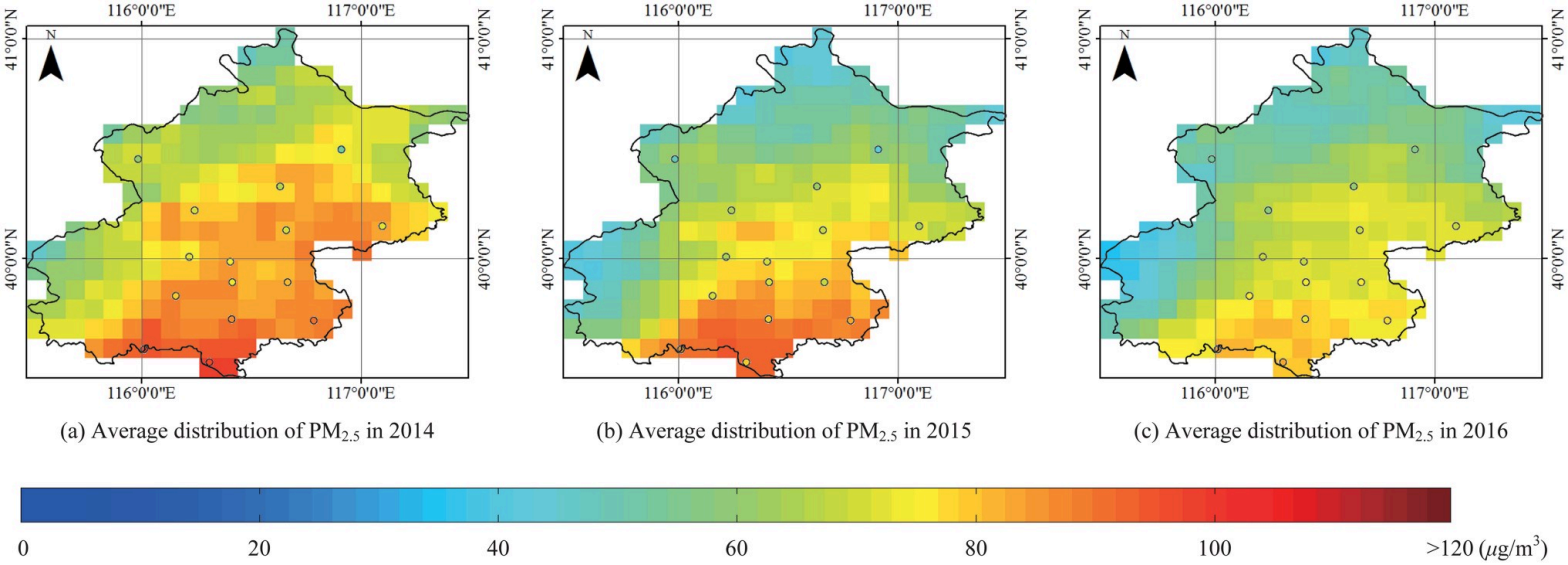
The annual average PM_2.5_ concentration distribution over Beijing during 2014 to 2016.

Fig 5 is the annual average PM_2.5_ concentration distribution over Beijing during 2014 to 2016. An obvious decrease of PM_2.5_ concentration can be intuitively found.

Although the satellite-derived PM_2.5_ monitoring method can provide larger spatial coverage than ground monitoring stations, the satellite has lower temporal coverage mainly due to bad observation conditions, such as clouds and fogginess. In our study, there were 477 model-valid days during 2014 to 2016. In addition, due to cloudy or foggy weather in Beijing giving rise to inefficient sampling frequency of available satellite observations, the AOD retrieval algorithm may be not valid. Thus other monitoring approaches with larger spatial coverage and smaller weather limitations should be developed.

Overall, the seasonal estimated PM_2.5_ concentration showed good consistency with those of ground measurements. We attempted to use relative humidity to correct MODIS AOD, but this brought little effect. Additional work should be done in this aspect.

## Conclusion

This paper focused on analysing the influence of BLH and *f*(RH) on the linear relationship between AOD and PM_2.5_ concentration and attempted to evaluate the improvement provided by modified regression models. As aerosol scale height, BLH can effectively improve the AOD-PM_2.5_ correlation by transforming AOD into near-surface aerosol extinction coefficient. When introduced NDVI, ST and SPD, the R^2^ in all seasons have about 2% increase. The spatial and temporal PM_2.5_ concentration distribution characteristics over Beijing during 2014 to 2016 were well revealed by seasonal and annual PM_2.5_ concentration distribution maps. The results confirmed the effectiveness of China’s clean air actions implemented from 2013 to 2017.

## Supporting information

S1 Table Geographical coordinates of 15 air quality monitoring stations used in this survey in Beijing. (DOCX)

## References

[1] Li X, Sun Y, An Y, Wang R, Lin H, Liu M, et al Air pollution during the winter period and respiratory tract microbial imbalance in a healthy young population in northeastern China. Environ Pollut. 2019;246:972–979. 10.1016/j.envpol.2018.12.083 31126003

[2] Dabass A, Talbott EO, Rager JR, Marsh GM, Venkat A, Holguin F, et al Systemic inflammatory markers associated with cardiovascular disease and acute and chronic exposure to fine particulate matter air pollution (PM_2.5_) among US NHANES adults with metabolic syndrome. Environ Res. 2018;161:485–491. 10.1016/j.envres.2017.11.042 29223110

[3] Lin C, Li Y, Yuan Z, Lau AK, Li C, Fung JC. Using satellite remote sensing data to estimate the high-resolution distribution of ground-level PM_2.5_. Remote Sens Environ. 2015; 156:117–128. 10.1016/j.rse.2014.09.015

[4] Huang T, Yu Y, Wei Y, Wang H, Huang W, Chen X. Spatial-seasonal characteristics and critical impact factors of PM2.5 concentration in the Beijing-Tianjin-Hebei urban agglomeration. PLoS ONE. 2018;13(9):e0201364 10.1371/journal.pone.0201364 30235240 PMC6147404

[5] Ma Z, Hu X, Huang L, Bi J, Liu Y. Estimating ground-level PM_2.5_ in China using satellite remote sensing. Environ Sci Technol. 2014; 48(13):7436–7444 10.1021/es5009399 24901806

[6] Zhao R, Gu X, Xue B, Zhang J, Ren W. Short period PM2.5 prediction based on multivariate linear regression model. PLoS ONE. 2018; 13(7): e0201011 10.1371/journal.pone.0201011 30048475 PMC6062037

[7] Griggs M. Measurements of atmospheric aerosol optical thickness over water using ERTS-1 data. J Air Pollut Control Assoc. 1975; 25(6):622–626. 10.1080/00022470.1975.10470118 1141544

[8] Butt MJ, Assiri ME, Ali MA. Assessment of AOD variability over Saudi Arabia using MODIS deep blue products. Environ Pollut. 2017; 231:143–153. 10.1016/j.envpol.2017.07.104 28800483

[9] Bilal M, Nichol JE. Evaluation of the NDVI-based pixel selection criteria of the MODIS C6 dark target and deep blue combined aerosol product. IEEE J Sel Top Appl Earth Observ Remote Sens. 2017; 10(8):3448–3453. 10.1109/JSTARS.2017.2693289

[10] Schäfer K, Harbusch A, Emeis S, Koepke P, Wiegner M. Correlation of aerosol mass near the ground with aerosol optical depth during two seasons in Munich. Atmos Environ. 2008; 42(18):4036–4046. 10.1016/j.atmosenv.2008.01.060

[11] Xin J, Zhang Q, Wang L, Gong C, Wang Y, Liu Z, et al The empirical relationship between the PM_2.5_ concentration and aerosol optical depth over the background of North China from 2009 to 2011. Atmos Res. 2014; 138:179–188. 10.1016/j.atmosres.2013.11.001

[12] Tai AP, Mickley LJ, Jacob DJ. Correlations between fine particulate matter (PM_2.5_) and meteorological variables in the United States: Implications for the sensitivity of PM2.5 to climate change. Atmos Environ. 2010; 44(32):3976–3984. 10.1016/j.atmosenv.2010.06.060

[13] Lee HJ, Liu Y, Coull BA, Schwartz J, Koutrakis P. A novel calibration approach of MODIS AOD data to predict PM_2.5_ concentrations. Atmos Chem Phys. 2011; 11(15):7991–8002. 10.5194/acp-11-7991-2011

[14] Xie Y, Wang Y, Zhang K, Dong W, Lv B, Bai Y. Daily estimation of ground-level PM_2.5_ concentrations over Beijing using 3 km resolution MODIS AOD. Environ Sci Technol. 2015; 49(20):12280–12288. 10.1021/acs.est.5b01413 26310776

[15] Zheng Y, Zhang Q, Liu Y, Geng G, He K. Estimating ground-level PM_2.5_ concentrations over three megalopolises in China using satellite-derived aerosol optical depth measurements. Atmos Environ. 2016; 124:232–242. 10.1016/j.atmosenv.2015.06.046

[16] You W, Zang Z, Zhang L, Li Y, Pan X, Wang W. National-scale estimates of ground-level PM_2.5_ concentration in China using geographically weighted regression based on 3 km resolution MODIS AOD. Remote Sens. 2016; 8(3):184 10.3390/rs8030184

[17] Guo Y, Tang Q, Gong DY, Zhang Z. Estimating ground-level PM_2.5_ concentrations in Beijing using a satellite-based geographically and temporally weighted regression model. Remote Sens Environ. 2017; 198:140–149. 10.1016/j.rse.2017.06.001

[18] Wang W, Mao F, Du L, Pan Z, Gong W, Fang S. Deriving hourly PM_2.5_ concentrations from Himawari-8 AODs over Beijing-Tianjin-Hebei in China. Remote Sens. 2017; 9(8):858 10.3390/rs9080858

[19] Zhang X, Hu H. Improving satellite-driven PM_2.5_ models with VIIRS Nighttime Light data in the Beijing-Tianjin-Hebei region, China. Remote Sens. 2017; 9(9):908 10.3390/rs9090908

[20] Eck TF, Holben BN, Reid JS, Xian P, Giles DM, Sinyuk A, et al Observations of the interaction and transport of fine mode aerosols with cloud and or fog in northeast asia from aerosol robotic network and satellite remote sensing. J Geophys Res-Atmos. 2018; 123:5560–5587. 10.1029/2018JD028313 32661496 PMC7356674

[21] Wei J, Li ZQ, Peng YR, Sun L. Modis collection 6.1 aerosol optical depth products over land and ocean: validation and comparison. Atmos Environ. 2019; 201:428–440. 10.1016/j.atmosenv.2018.12.004

[22] Wang Q, Zeng Q, Tao J, Sun L, Zhang L, Gu T, et al Estimating PM_2.5_ concentrations based on MODIS AOD and NAQPMS data over Beijing-Tianjin-Hebei. Sensors. 2019; 19(5):1207 10.3390/s19051207 PMC6427133 30857313

[23] You W, Zang ZL, Pan XB, Zhang LF, Chen D. Estimating PM_2.5_ in Xi’an, China using aerosol optical depth: A comparison between the MODIS and MISR retrieval models. Sci Total Environ. 2015; 505:1156–1165. 10.1016/j.scitotenv.2014.11.024 25466686

[24] Geng G, Zhang Q, Martin RV, Donkelaar A, Huo H, Che H, et al Estimating long-term PM_2.5_ concentrations in China using satellite-based aerosol optical depth and a chemical transport model. Remote Sens Environ. 2015; 166:262–270. 10.1016/j.rse.2015.05.016

[25] Hoff RM, Christopher SA. Remote sensing of particulate pollution from space: have we reached the promised land? J Air Waste Manage Assoc. 2009; 59(6):645–675. 10.3155/1047-3289.59.6.645 19603734

[26] Koelemeijer RBA, Homan CD, Matthijsen J. Comparison of spatial and temporal variations of aerosol optical thickness and particulate matter over Europe. Atmos Environ. 2006; 40(27):5304–5315. 10.1016/j.atmosenv.2006.04.044

[27] Guo YJ, Feng N, Christopher SA, Kang P, Zhan FB, Hong S. Satellite remote sensing of fine particulate matter (PM_2.5_) air quality over Beijing using MODIS. Int J Remote Sens. 2014; 35(17):6522–6544. 10.1080/01431161.2014.958245

[28] Tsai TC, Jeng YJ, Chu DA, Chen JP, Chang SC. Analysis of the relationship between MODIS aerosol optical depth and particulate matter from 2006 to 2008. Atmos Environ. 2011; 45(27):4777–4788. 10.1016/j.atmosenv.2009.10.006

[29] Q, Zheng YX, Tong D, Shao M, Wang SX, Zhang YH, et al Drivers of improved PM2.5 air quality in China from 2013 to 2017. P NATL ACAD SCI USA. 2019; 116(49):24463–24469. 10.1073/pnas.1907956116 PMC6900509 31740599

