# Supplementary material for: Estimation of ground-level PM2.5 concentration using MODIS AOD and corrected regression model over Beijing, China
Source: PLoS One. 2020 Oct 13;15(10):e0240430. doi: 10.1371/journal.pone.0240430 (PMC7553281; doi:10.1371/journal.pone.0240430)
Supplement: S1 Table — (DOCX) [file pone.0240430.s001.docx]

**S1 Table. Geographical coordinates of 15 air quality monitoring stations in Beijing.**

| **No.** | **Longitude (E)** | **Latitude (N)** |
| --- | --- | --- |
| 1 | 116.417° | 39.886° |
| 2 | 116.397 | 39.982° |
| 3 | 116.207° | 40.002° |
| 4 | 116.146° | 39.824° |
| 5 | 116.404° | 39.718° |
| 6 | 116.663° | 39.886° |
| 7 | 116.655° | 40.127° |
| 8 | 116.23° | 40.217° |
| 9 | 117.1° | 40.143° |
| 10 | 116.628° | 40.328° |
| 11 | 115.972° | 40.453° |
| 12 | 116.911° | 40.499° |
| 13 | 116.783° | 39.712° |
| 14 | 116.30° | 39.52° |
| 15 | 116.00° | 39.58° |
